# Efficient treatment of breast cancer xenografts with multifunctionalized iron oxide nanoparticles combining magnetic hyperthermia and anti-cancer drug delivery

**DOI:** 10.1186/s13058-015-0576-1

**Published:** 2015-05-13

**Authors:** Susanne Kossatz, Julia Grandke, Pierre Couleaud, Alfonso Latorre, Antonio Aires, Kieran Crosbie-Staunton, Robert Ludwig, Heidi Dähring, Volker Ettelt, Ana Lazaro-Carrillo, Macarena Calero, Maha Sader, José Courty, Yuri Volkov, Adriele Prina-Mello, Angeles Villanueva, Álvaro Somoza, Aitziber L Cortajarena, Rodolfo Miranda, Ingrid Hilger

**Affiliations:** Institute for Diagnostic and Interventional Radiology, Jena University Hospital – Friedrich Schiller University Jena, D-07740 Jena, Germany; Instituto Madrileño de Estudios Avanzados en Nanociencia (IMDEA Nanociencia), Campus Universitario de Cantoblanco, 28049 Madrid, Spain; Unidad Asociada de Nanobiotecnología CNB-CSIC & IMDEA Nanociencia, Campus Universitario de Cantoblanco, 28049 Madrid, Spain; School of Medicine, Trinity College Dublin, Dublin, Ireland; Departamento de Biología, Universidad Autónoma de Madrid, Cantoblanco, 28049 Madrid, Spain; Laboratoire CRRET, Université Paris EST Créteil, 61 Avenue du Général de Gaulle, 94010 Créteil, France; CRANN, Trinity College, Dublin, Ireland

## Abstract

**Introduction:**

Tumor cells can effectively be killed by heat, e.g. by using magnetic hyperthermia. The main challenge in the field, however, is the generation of therapeutic temperatures selectively in the whole tumor region. We aimed to improve magnetic hyperthermia of breast cancer by using innovative nanoparticles which display a high heating potential and are functionalized with a cell internalization and a chemotherapeutic agent to increase cell death.

**Methods:**

The superparamagnetic iron oxide nanoparticles (MF66) were electrostatically functionalized with either Nucant multivalent pseudopeptide (N6L; MF66-N6L), doxorubicin (DOX; MF66-DOX) or both (MF66-N6LDOX). Their cytotoxic potential was assessed in a breast adenocarcinoma cell line MDA-MB-231. Therapeutic efficacy was analyzed on subcutaneous MDA-MB-231 tumor bearing female athymic nude mice.

**Results:**

All nanoparticle variants showed an excellent heating potential around 500 W/g Fe in the alternating magnetic field (AMF, conditions: *H* = 15.4 kA/m, *f* = 435 kHz). We could show a gradual inter- and intracellular release of the ligands, and nanoparticle uptake in cells was increased by the N6L functionalization. MF66-DOX and MF66-N6LDOX in combination with hyperthermia were more cytotoxic to breast cancer cells than the respective free ligands. We observed a substantial tumor growth inhibition (to 40% of the initial tumor volume, complete tumor regression in many cases) after intratumoral injection of the nanoparticles *in vivo*. The proliferative activity of the remaining tumor tissue was distinctly reduced.

**Conclusion:**

The therapeutic effects of breast cancer magnetic hyperthermia could be strongly enhanced by the combination of MF66 functionalized with N6L and DOX and magnetic hyperthermia. Our approach combines two ways of tumor cell killing (magnetic hyperthermia and chemotherapy) and represents a straightforward strategy for translation into the clinical practice when injecting nanoparticles intratumorally.

**Electronic supplementary material:**

The online version of this article (doi:10.1186/s13058-015-0576-1) contains supplementary material, which is available to authorized users.

## Introduction

Nanoparticles are extensively investigated in the field of nanomedicine because they can be utilized in a wide range of applications, such as drug delivery, disease imaging and therapy. Functionalization of nanoparticles with cytotoxic drugs or tumor-specific proteins has been proven a promising technique, especially in cancer research, to selectively target tumor cells, improve drug delivery and reduce systemic toxicity of drugs [1–5]. A defined subgroup of nanoparticles, particularly the superparamagnetic ones (MNP), can release heat during the exposure to an alternating magnetic field (AMF) in order to kill tumor cells with hyperthermic temperatures [6–8]. This so-called magnetic hyperthermia approach has also yielded encouraging results in preclinical research [9–11]. Translation into clinical practice is particularly expected for intratumoral injection of the magnetic material [9]. The advantages are that MNP are directly deposited at the target site and the amount of MNP can be selectively modulated based on tumor size. For intravenous application, quantities of MNP higher than physiological levels, that might even be cytotoxic, need to be injected [12]. Here, the biggest challenge is selective accumulation within the tumor, which can be prevented by the intratumoral injection.

Adding biologically active functionalization to the MNP bears great potential to further refine and improve magnetic hyperthermia therapy [13, 14]. Among them, the nucleolin antagonist multivalent pseudopeptide Nucant (N6L), which is currently being applied in a phase II clinical trial, is of particular interest. This molecule targets a nucleolin-receptor complex overexpressed selectively at the cell surface of tumor cells, and mediates lethal effects to cancer cells after internalization [15–17], and antitumor activities [18].

Furthermore, the chemotherapeutic drug doxorubicin (DOX) is currently used in clinical cancer therapy. However, to reduce its systemic toxicity and side effects, this drug is constantly under investigation to be used in a drug carrier system that can be activated (e.g. by heat or pH-sensitive liposomes) [19–23]. Functionalization of DOX to iron oxide nanoparticles is a straightforward alternative, because the release, and therefore the activation of DOX can be triggered by magnetically induced heating [24], particularly if the biologically active substances N6L and DOX [25] are electrostatically bound to the nanoparticles. Thus, upon change of the ionic strength or the pH, the cargo molecules will be released in the close vicinity or inside the target cells after intratumoral application of the magnetic material.

Here, we propose a strategy to improve the performance and the outcome of heat treatment for tumors by magnetic hyperthermia, using newly developed MNP that are functionalized through electrostatic interaction. By using a tumor-specific cell internalization moiety (N6L) and/or an anti-cancer drug (DOX) on the MNP surface, we aim at enhancing the intracellular MNP uptake as well as mediating cytotoxic effects beyond hyperthermia, to reach tumor cells that escape the heat treatment. To the best of our knowledge, we developed a novel combination therapy based on multifunctionalized MNP to selectively target and successfully eliminate breast cancer cells.

## Methods

### Nanoparticle synthesis

The MNP used in this study, denominated MF66, were produced by means of the co-precipitation technique [26]. Coating with dimercaptosuccinic acid (DMSA) was performed as described previously [27, 28]. Briefly, the MNP were initially coated with oleic acid and dispersed in toluene and a solution of DMSA in dimethyl sulfoxide (DMSO) was added to perform a ligand exchange from oleic acid to DMSA. The DMSA-coated MNP precipitated and were washed several times with water. Finally, the MNP were resuspended in distilled water, the pH adjusted and sterile filtration carried out. The MF66 MNP used in this study are already characterized and their magnetic properties have been studied [28]. The hydrodynamic diameter was measured by dynamic light scattering (DLS) and expressed as the Z-average size of the MNP dispersed in water. Furthermore, we measured the ζ- potential (Zetasizer Nano ZS, Malvern Instruments) of the MNP at pH 7.4.

### MNP functionalization

For the electrostatic immobilization, either 5.1 μl of an N6L solution (1.93 mM) or 202 μl of a DOX hydrochloride solution (500 μM, Cell pharm, Bad Vilbel, Germany) were incubated with 1 ml MF66 for 6 h at room temperature. N6L multivalent pseudopeptide was synthesized, purified and analyzed for its biological effect as previously described [18]. In the case of N6L, the mixture was purified by ultrafiltration or centrifuged. MF66-DOX was centrifuged and the supernatant was removed. MNP pellets were re-dispersed at 2.4 mg Fe/ml.

To immobilize both DOX and N6L onto MF66 MNP, the same protocol as described above was used; here, DOX was immobilized first onto MF66 MNP followed by N6L. To quantify the amount of immobilized N6L, an N6L fluorescently labeled with Alexa Fluor 546 (N6L-AF546) was synthesized and used for immobilization onto MF66 under the same conditions as described for N6L. The unbound N6L-AF546 recovered during the washes was measured (λ_exc_ = 555 nm, λ_em_ = 560–750 nm). Similarly, immobilized DOX was quantified by measuring the fluorescence of unbound DOX in the supernatant (λ_exc_ = 495 nm, λ_em_ = 520–750 nm).

For the preparation of the MNPs used for the in vitro and in vivo studies, the bare MNPs were sonicated for 3 minutes and then filtered through a 0.22-μm strainer for sterilization. The iron concentration was measured after filtration by Inductively coupled plasma – mass spectrometry (ICP-MS) before functionalization. All functionalization processes were then carried out under sterile conditions and all molecules used (N6L or DOX solutions) were filtered through a 0.22-μm strainer.

### Specific absorption rate of MNP

To assess the heating potential of the MNP in an AMF (conditions *H* = 15.4 kA/m, *f* = 435 kHz) we determined the specific absorption rate (SAR) and intrinsic loss power (ILP) using colorimetric methods as described before [28].

### Release of DOX and N6L-AF546

The mode of the release of the electrostatically immobilized molecules onto MNP was monitored [13]. Water was used to determine the stability of the three formulations over time. Then, the same experiments were performed in PBS buffer (pH 7.4) and in phenol red-free DMEM with 10% (v/v) fetal bovine serum (FBS) (complete DMEM) to modulate desorption of N6L-AF546 and DOX from the MNP in the presence of salts. MNP were dispersed at a final concentration of 0.3 mg Fe/ml. The samples were then placed at 37°C and at different time points (up to 120 h), 100 μl of each sample were collected, centrifuged and supernatants were analyzed by fluorescence and compared to a reference sample.

### Cell culture

Three genotypically diverse breast-derived cell lines were used for in vitro testing of the MNP. Two cell lines (MCF-7 and MDA-MB-231, both ATCC) were selected due to their distinct cancer phenotypes. In comparison to the two cancerous cell line models, a third non-cancerous cell line MCF-10A (ATCC, mammary epithelial cells) was used as a control. Cell lines were cultured at 37°C in a humidified atmosphere containing 5% CO_2_ and maintained in DMEM with 10% (v/v) FBS and 1% PenStrep (all products from Gibco®, Paisley, Scotland, UK). Cells were tested regularly using the MycoAlert® PLUS test kit (Lonza, Switzerland) for the presence of mycoplasma and prior to freezing stock. All experiments were conducted using sub-confluent cells in the exponential phase of growth. Depending on the experiment, cells were seeded in 24-well or 96-well plates and incubated for 24 h prior to MNP exposure.

### MNP internalization and subcellular localization

MDA-MB-231 cells grown on coverslips were incubated for 24 h with MF66, MF66-N6L, MF66-DOX or MF66-N6LDOX (all at 100 μg Fe/ml) in cell culture medium. To remove non-internalized MNP, samples were washed and observed immediately or after 48 h, under bright light for internalization, or by fluorescence microscopy for subcellular location of DOX (n = 3 independent experiments).

### Prussian blue staining for iron detection

The presence and localization of iron particles in MDA-MB-231 cells were assessed by Prussian blue staining. Cells were incubated with MNP for 24 h and then analyzed immediately or 48 h post incubation. Cells were washed, fixed in methanol, and stained with equal volumes of 4% hydrochloric acid and 4% potassium ferrocyanide trihydrate (all Panreac Química) for 15 minutes, and counterstained with neutral red.

### Impact of nanoparticles on cells in the absence of hyperthermic conditions

Cells were allowed to attach to the culture plate for 24 h and then exposed for 24 h and 72 h to the MNP formulations. Concentrations in the range of 5 to 200 μg Fe/mL were employed to determine if the selected MNP formulation elicited a cytotoxic response in each cell line. Triplicate experiments were conducted with three wells per concentration. Positive and negative controls were included as previously described [29]. Following 24 h incubation, cells were washed, stained for 30 minutes using LysoTracker® (Molecular Probes, Eugene, OR, USA), an indicator for cell membrane permeability, fixed using 3.7% paraformaldehyde (PFA) for 20 minutes and further stained for 10 minutes with Hoechst 33342 nuclear dye (Thermo Fisher Scientific Inc., Waltham, MA, USA). Screening was carried out by high content analysis using the GE Healthcare InCell1000 Analyzer, Buckinghamshire, UK by bright field and three fluorescent channels as described before [29].

### In vitro cell viability determination under hyperthermic conditions

The sensitivity of MDA-MB-231 cells to hyperthermic temperatures and the effect of the MNP formulations on cell viability in the presence and absence of heat were assessed. At 24 h after seeding 5000 cells/well in a 96-well plate (Greiner BioOne, Frickenhausen, Germany), cells were incubated with either medium, MF66, MF66-N6L, MF66-DOX or MF66-N6LDOX in a concentration of 100 μg Fe/ml or the equivalent molar amount of free N6L (400 nM), DOX (4 μM) or N6L and DOX for 24 h at 37°C.

For hyperthermia treatment, cells were put in the incubator at 46°C for 30 minutes, corresponding to a temperature dosage of 90 cumulative equivalent minutes at 43°C (CEM43T90, for details see Additional file 1), or else cells were left in the incubator at 37°C. At 48 h after hyperthermia, cells were washed twice with Hank's balanced salt solution (HBSS), once with medium and then AlamarBlue® reagent was added for 4 h [30]. Fluorescence was measured (Tecan Infinite M1000 Pro, Grödig, Austria; λ_exc_ = 530–560 nm, λ_em_ = 590 nm) and normalized to the fluorescence of untreated cells (no MNP incubation and no hyperthermia).

### Hyperthermia treatment in tumor-bearing athymic nude mice

All in vivo hyperthermia experiments, the experimental workflow, determination of temperature dosage and heat distribution and data analysis were carried out as described before in detail [28]. All experiments were in accordance with international guidelines on the ethical use of animals and were approved by the regional animal care committee (02-068/11, Thüringer Landesamt für Verbraucherschutz, Bad Langensalza, Germany). Animals were maintained under artificial day-night cycles (12 h light-dark cycles; 23°C room temperature, 30%–60% environment humidity) and received food and water ad libitum.

**Figure 1:**
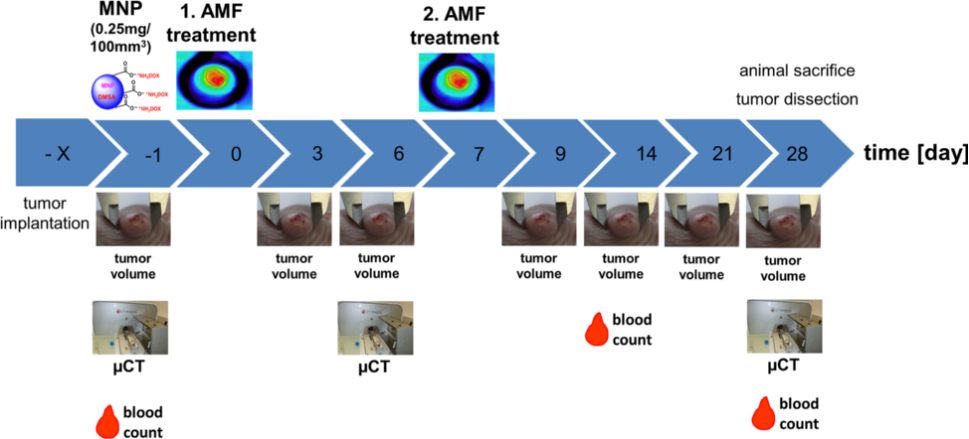
Experimental workflow for magnetic hyperthermia in vivo. After tumor implantation, magnetic nanoparticles (MNP) were applied intratumorally 24 h prior to the first magnetic hyperthermia treatment (60 minutes, alternating magnetic field (AMF) *H* = 15.5 kA m^−1^, *f* = 435 kHz). Seven days later a second hyperthermia treatment was performed. Tumor volume, blood count and MNP distribution were monitored by micro computed tomography (μCT) on the indicated days. After the experimental period of 28 days the animals were sacrificied and the tumors dissected.

Briefly, to induce xenografts we injected 120 μl Matrigel™ containing 2 × 10^6^ MDA-MB-231 cells subcutaneously on the rear backside of the nude mice and allowed tumor growth until a volume between 100 and 250 mm^3^ was reached. MNP formulations MF66, MF66-N6L, MF66-DOX or MF66-N6LDOX were used in concentrations of 0.25 mg Fe/100 mm^3^ for intratumoral injection 24 h prior to the first in vivo hyperthermia treatment (Figure 1). Depending on the tumor size this equaled a concentration range of 0.15–0.375 mg N6L/kg body weight and 0.22–0.55 mg DOX/kg body weight. Hyperthermia treatment were conducted on days 0 and 7. Tumor volume was measured with a caliper every 3 days and compared to the untreated control animals (ddH_2_O injection, no AMF treatment). The tumors of anesthetized animals were placed inside the coil of the AMF (*H* = 15.4 kA/m, *f* = 435 kHz) for magnetic hyperthermia treatment. The tumor surface and rectal temperatures were monitored by fiber optic temperature sensors. To ensure animal safety, we ensured that the temperatures in non-tumor tissue and the rectum did not exceed 38°C during hyperthermia treatment. We calculated the temperature dosage on the tumor surface, namely T90 temperatures and CEM43T90 values according to Sapareto et al. [31] based on the infrared thermography data. To determine complete tumor regression, a relative tumor volume of 20% was chosen as the cutoff value to account for remaining skin and cicatrical tissue.

### Micro computed tomography (µCT) imaging of intratumoral MNP distribution

For optimization of the applied heat distribution on tumors, we analyzed the individual intratumoral MNP distribution during in vivo hyperthermia treatment. Therefore, we conducted µCT imaging of the animals directly after MNP application and for follow up on days 7 and 28 (TomoScope Synergy Twin, CT Imaging, Erlangen, Germany) using a low radiation-dose protocol (29 s, 65 kV). MNP distribution and volume were analyzed with the Imalytics Research Software (Philips Technologie, Aachen, Germany). Using these data, the AMF power was controlled individually to reach 43°C in tumor areas farthest away from MNP deposits.

### Iron determination in organs

To investigate MNP biodistribution and degradation, the iron content of tumors and organs was quantified using flame atomic absorption spectroscopy as previously described [28].

### Histology

The proliferative behavior of cells depending on magnetic hyperthermia treatment and the MNP formulation was investigated at day 28 after the first magnetic hyperthermia treatment by assessing the Ki67 and Bcl2 protein abundance in paraffin-embedded tissue sections. The degree of vascularization was assessed by CD31 staining. The primary antibodies used were a monoclonal anti-Ki67 antibody (Abcam, Cambridge, UK, 1:500 dilution), a monoclonal mouse anti-human Bcl2 (1:500 dilution, Dako, Hamburg, Germany) and a polyclonal rabbit anti-CD31 (1:500 dilution, Abcam, Cambridge, UK). Antigen detection was visualized via streptavidin-alkaline phosphatase or horseradish peroxidase (for details of staining protocols see Additional file 1). The slides were evaluated by three blinded observers. Ki67/Bcl2-positive areas over the whole tumor sections were evaluated and divided into five categories based on their expression level: (I) no expression to (V) very high expression as described before [28].

### Statistics

Statistical analysis of cell staining with LysoTracker® was performed as previously described for multiparametric assessment [29]. To determine if differences of tumor volume between animal groups were significant, we conducted the Mann-Whitney U test for analysis of tumor volume and one-way analysis of variance (ANOVA) for in vitro data analysis. For histological differences the Mann-Whitney U test was conducted for MNP with and without hyperthermia, and for comparison between the different MNP based on the expression level. The significance level was set at *p* ≤0.05.

## Results

### Functionalized nanoparticles with high heating potential

**Figure 2:**
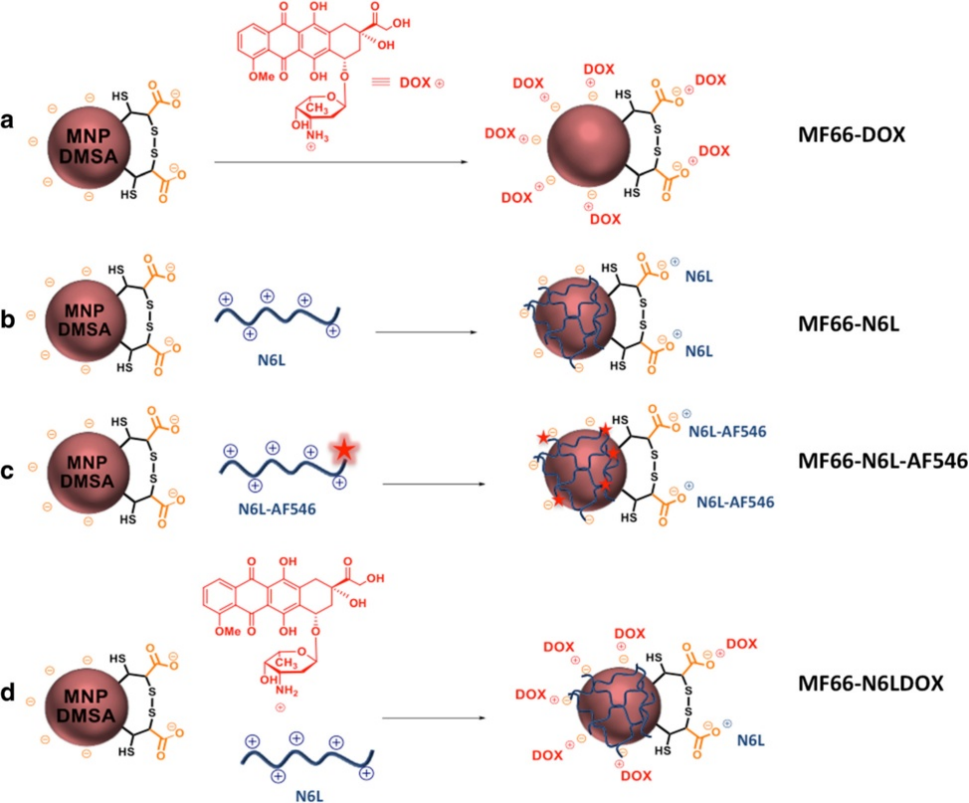
Design of the functionalized magnetic nanoparticles (MNP). **a** The maximum amount of doxorubicin (DOX) loaded electrostatically, keeping the colloidal stability of the MF66 MNP, was 40 μmol/g Fe. MF66, redispersed at 2.4 mg Fe/ml, gave a final concentration of DOX of 96 μM. **b** The maximum amount of Nucant multivalent pseudopeptide (N6L) loaded electrostatically, keeping the colloidal stability of the MF66, was 4 μmol/g Fe. **c** To quantify the amount of N6L effectively adsorbed onto the MF66, N6L-AF546 fluorescent variant was used to monitor the unbound N6L-AF546 recovered in the washes by fluorescence. When 4.1 μmol N6L-AF546 per 1 g Fe was added the yield of immobilization was above 98%, obtaining a final amount of 4 μmol N6L-AF546/g Fe. **d** Multifunctionalization of MF66 was achieved following a sequential procedure. First, DOX was immobilized onto the surface of MF66 using the same protocol described above. Then, N6L was added over MF66-DOX leading to the formation of bifunctional MNP. Multifunctionalized MNP with both DOX and N6L were re-dispersed at 2.4 mg Fe/ml leading to concentrations of DOX = 96 μM and N6L = 9.6 μM.

The MF66 MNP, consisting of an iron oxide core of 12 ± 3 nm (magnetite) coated with DMSA [28], were successfully functionalized with N6L, DOX or both. The loading of MF66 with the N6L peptide was estimated using a derivate labeled with a fluorescent dye (N6L-AF546). Thus, using the nanoparticle MF66-N6L-AF546 we were able to quantify the unbound peptide and calculate the immobilization yield (98%). This approach produced stable MNP loaded with 4 μmol N6L/g Fe (9.6 μM N6L at 2.4 mg Fe/ml). In the case of MF66-DOX, the yield of immobilization was 95% DOX leading to stable MNP loaded with 40 μmol DOX/g Fe (96 μM DOX at 2.4 mg Fe/ml) (Figure 2). This equals a mass ratio of 40/60 N6L/DOX and a molar ratio of 1/10 N6L/DOX based on the immobilization of 0.015 g N6L and/or 0.022 g DOX on 1 g of Fe. In water the dispersions were stable for at least 10 days with a uniform size distribution (Additional file 2: Figure S1a). In complete DMEM a protein corona was formed and led to an increased hydrodynamic size; however, the MNP did not fully agglomerate and were stable for at least 48 h (Additional file 2: Figure S1b).

**Figure 3:**
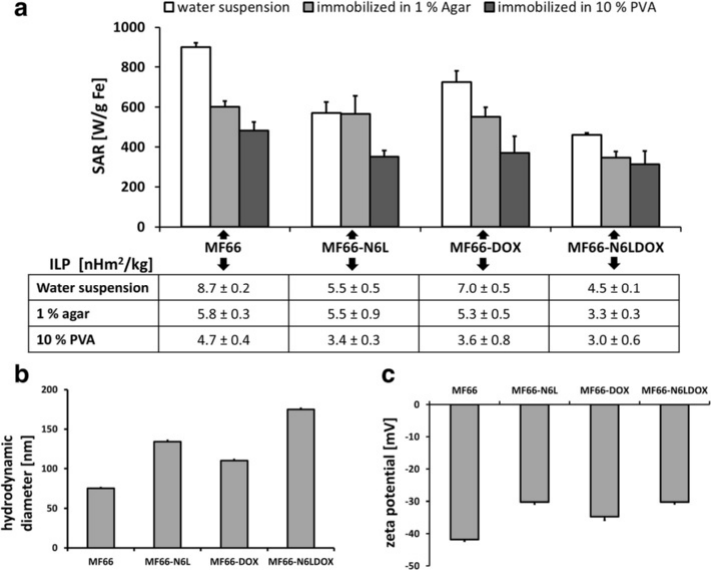
High heating potential of magnetic nanoparticles (MNP) in conjunction with moderate hydrodynamic size and negative ζ-potential. Specific absorption rate (SAR) and intrinsic loss power (ILP) values of MF66-N6L, MF66-DOX, MF66-N6LDOX and the non-functionalized variant MF66 after suspension in different media (water, 1% (w/v) agar in water, 10% PVA in dimethyl sulfoxide (DMSO)/water (80/20% (v/v)). **a** Immersion in agar and PVA mimics different degrees of MNP immobilization, as it occurs after uptake in tumor tissue. Values of hydrodynamic size (z-average) **(b)** and ζ-potential **(c)** are also displayed for the three functionalized MNP formulations and for the non-functionalized variant.

One of the decisive characteristics of nanoparticles is their potential to absorb energy and release heat, which is lethal to tumor cells. The investigated MNP (water suspension) displayed high SAR values between 400 and 700 W/g Fe (*H* = 15.4 kA/m, *f* = 435 kHz), corresponding to ILPs between 4.5 and 7 nHm^2^/kg (Figure 3a). Without functionalization, MF66 displayed a SAR of 900 ± 22 W/g Fe and an ILP of 8.7 ± 0.2 nHm^2^/kg, respectively. Under different degrees of immobilization in agarose and a Polyvinyl alcohol (PVA) hydrogel, where Brownian motion of the MNP is inhibited, the SAR was reduced. Interestingly, the largest SAR reduction of 50% occurred in the MF66-DOX, which had the highest SAR before immobilization, resulting in comparable SAR values between 314 and 370 W/g Fe (ILP 3.0–3.6 nHm^2^/kg) of all MNP formulations after immobilization in PVA.

The hydrodynamic diameter was increased in MF66-N6LDOX (175 nm) compared to MF66-N6L (134 nm), MF66-DOX (110 nm) and bare MF66 (75 nm) (Figure 3b). The functionalized MNP displayed a less negative ζ-potential compared to bare MF66 (−41.8 ± 0.3 mV at pH 7). MF66-DOX had a ζ-potential of −34.7 ± 0.6 mV at pH 7.4, MF66-N6L displayed −30.2 ± 1.1 mV at pH 7.2 and MF66-N6LDOX had a surface charge of −30.2 ± 0.5 at pH 7.2 (Figure 3c).

### N6L-AF546 and DOX are released in different biological media

**Figure 4:**
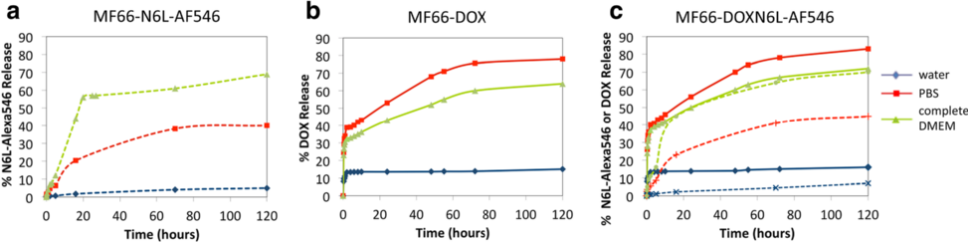
Nucant multivalent pseudopeptide labeled with Alexa Fluor 546 (*N6L-AF546*) and doxorubicin (*DOX*) were slowly released from magnetic nanoparticles (MNP) in the presence of salts. Release behavior of the electrostatically immobilized molecules was studied by dispersing: **a** MF66-N6L-AF546, **b** MF66-DOX, **c** MF66-DOXN6L-AF546 in water (blue), PBS buffer (pH 7.4) (red) or phenol-red-free complete DMEM (green). *Plain lines* represent the release of DOX and *dashed lines* represent the release of N6L-AF546. Samples were incubated at 37°C and at different times supernatants were analyzed by fluorescence (Dox λ_exc_ = 495 nm, λ_em_ = 600 nm and N6L-AF546 λ_exc_ = 555 nm, λ_em_ = 575 nm; for MF66-DOXN6L-AF546 each emission signal was corrected for the fluorescence of the other immobilized molecule). Values were compared to a reference sample containing the total amount of the corresponding molecule immobilized on the MNP.

The use of the electrostatic conjugations for in vitro and in vivo application was assessed by quantifying the release of N6L-AF546 and DOX molecules from the MF66 functionalized with fluorescently labeled N6L (MF66-N6L-AF546), with DOX (MF66-DOX) or with both molecules (MF66-DOXN6L-AF546). In PBS and complete DMEM, N6L-AF546 and DOX were released in relatively high concentrations due to the presence of salts and biomolecules in these media. In both media, the release stopped after around 80 h of incubation (Figure 4). At this point, 40% in PBS or 69% in complete DMEM of the total conjugated N6L-AF546 was released. For the DOX functionalization, the maximal release corresponded to 78% in PBS and 64% in complete DMEM of the total conjugated DOX. MF66-DOXN6L-AF546 showed similar releases of both immobilized molecules (N6L-AF546 and DOX) as the ones observed for MF66-N6L-AF546 or MF66-DOX. In water, the storage condition for the nanoparticles, the release of N6L-AF546 and DOX functionalization was very low (below 15% of the total conjugated amount), highlighting the overall stability of the molecules bound to the MF66 MNP.

### N6L enhanced MNP internalization and DOX diffused into the nucleus

**Figure 5:**
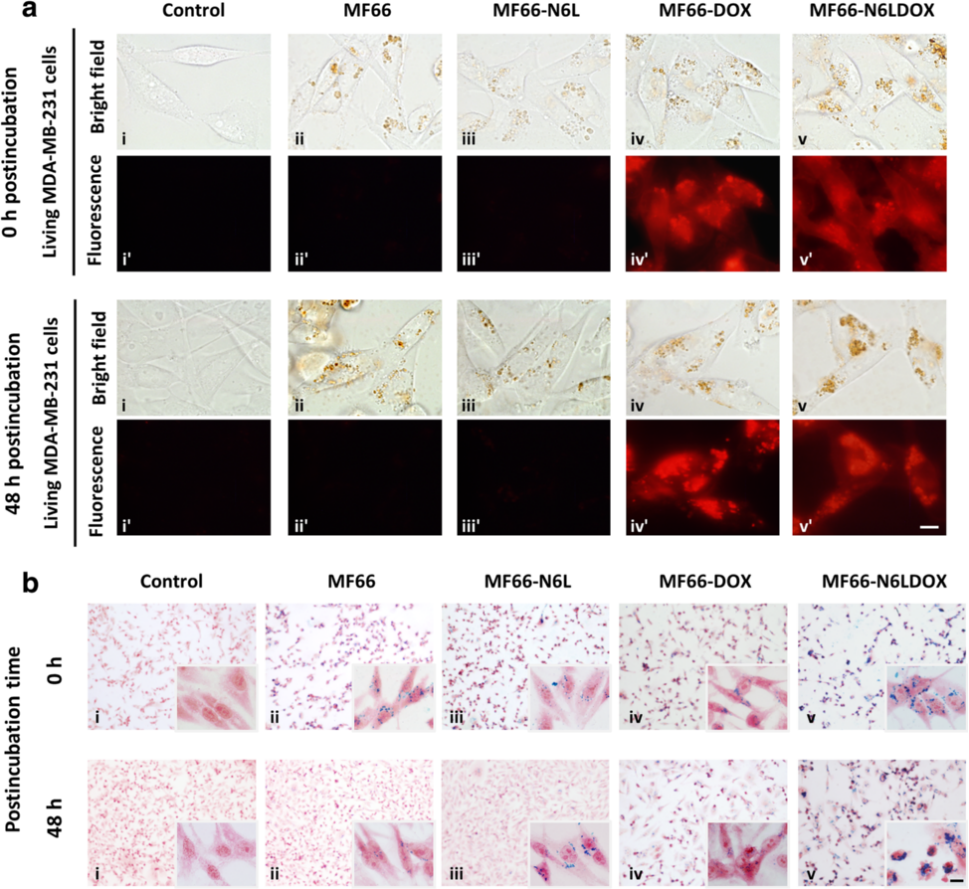
Magnetic nanoparticle (*MNP*) uptake depends on functionalization and Nucant multivalent pseudopeptide (*N6L*) and doxorubicin (*DOX*) were slowly released intracellularly. Cell uptake of the different MNP formulations (MF66, MF66-N6L, MF66-DOX or MF66-N6LDOX) in living MDA-MB-231 cells incubated with MNP at 100 μg Fe/ml for 24 h visualized on optical microscopy immediately (0 h) or 48 h post incubation (Olympus BX61 epifluorescence microscope equipped with an Olympus DP50 digital camera (Olympus, Tokyo, Japan), and processed using Photoshop CS2 software (Adobe Systems, San Jose, CA, USA)). **a** Live cells were imaged under bright field (MNP, *brown spots*) and fluorescence microcopy (DOX, *red emission*). **i**, **i’** Untreated control cells. **ii**, **ii’** Cells incubated with MF66 MNP. **iii**, **iii’** Cells incubated with MF66-N6L. **iv**, **iv’** Cells incubated with MF66-DOX. **v**, **v’** Cells incubated with MF66-N6LDOX. **b** Internalization and uptake of MNP (*blue spots*) inside MDA-MB-231 cells stained with Prussian blue reaction for iron oxide detection after 0 and 48 h post incubation. **i** Untreated control cells. **ii** Cells incubated with MF66 MNP. **iii** Cells incubated with MF66-N6L. **iv** Cells incubated with MF66-DOX. **v** Cells incubated with MF66-N6LDOX. Scale bar = 10 μm.

After 24 h incubation of MDA-MB-231 cells with bare MF66 MNP, a substantial fraction of internalized MF66 MNP were accumulated in the lysosomal compartment of cells (Additional file 3: Figure S2). However, 0 h or 48 h post-incubation with MF66-DOX and MF66-N6LDOX, we detected DOX not only in lysosomes (colocalization with the brown colored MNP in the bright field images; Figure 5a), but also in cytoplasm and nuclei (diffuse red fluorescence in fluorescence images; Figure 5a). These results strongly suggest that DOX molecules gradually diffused into nuclei, a necessary condition to induce DNA damage. MDA-MB-231 cells incubated with MF66 and MF66-N6L and control cells did not show any appreciable red fluorescence. We confirmed that MF66, monofunctionalized and multifunctionalized MF66 were efficiently internalized into MDA-MB-231 cells by Prussian blue staining (specific for iron detection). Furthermore, nanoparticles within cells were still observed 48 h after incubation. As shown in Figure 5b, immediately after incubation cells showed an elongated shape and were partially superimposed on each other, similar to control cells (non-treated), indicating that MNP accumulation into the cells did not induce cytotoxic effects. On the contrary, 48 h after treatment with MF66-DOX or MF66-N6LDOX we detected a decrease in cell number compared to their respective non-treated controls. In addition, in both samples, but especially in MF66-N6LDOX, we visualized cells with clear apoptotic morphology, including cell shrinkage and condensed and fragmented nuclei (magnified images 5b iv and v). On the other hand, we also observed higher amounts of MF66-N6L and MF66-N6LDOX internalized and still retained at 48 h post incubation, compared to MF66 and MF66-DOX. These results indicate that N6L enhanced cellular uptake of MNP (Figure 5b).

### Cytotoxic effects of MNPs in vitro were more pronounced in combination with hyperthermia

**Figure 6:**
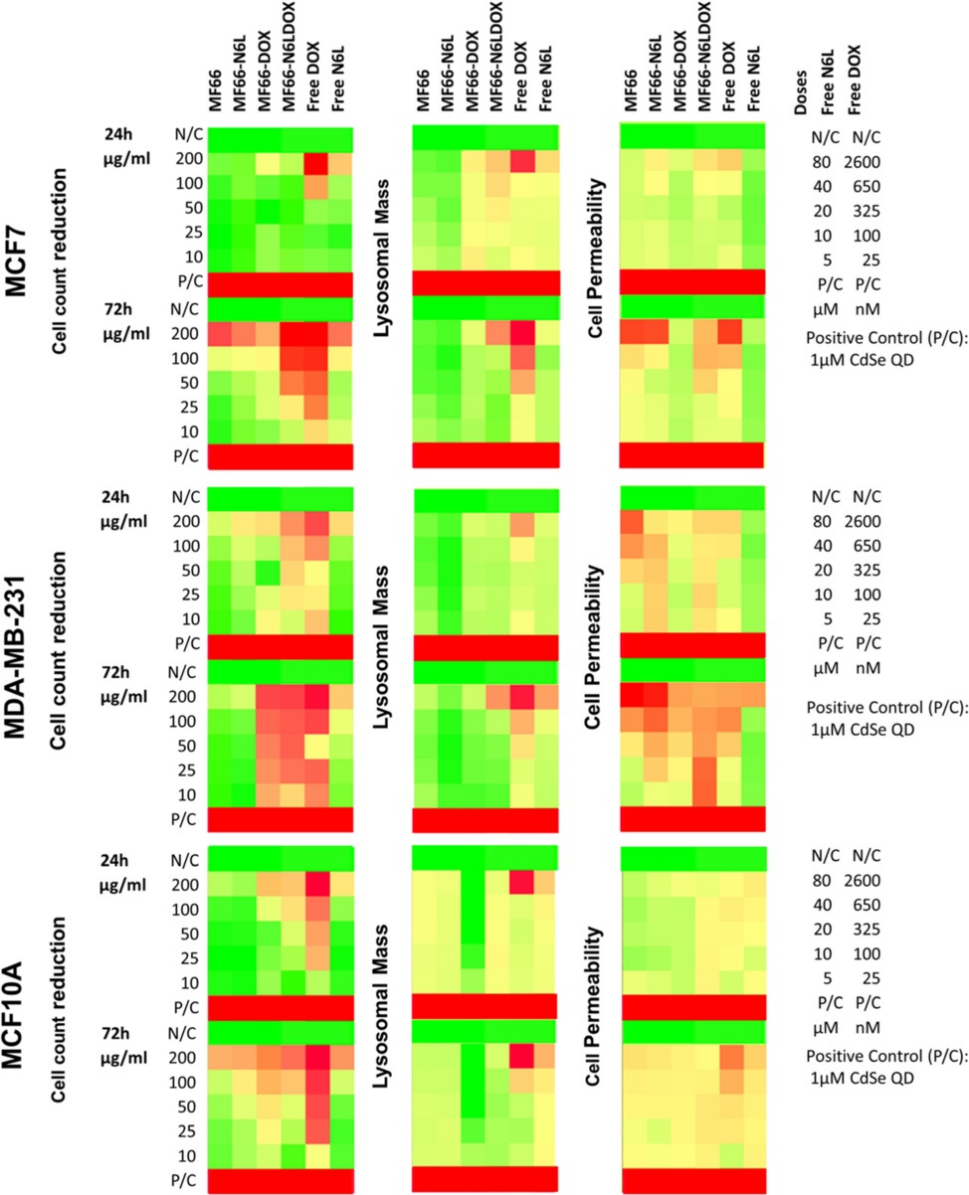
Color-coded map of multiparametric cytotoxicity evaluation in three breast cell lines (two with distinct cancer phenotypes (*MCF7* and *MDA-MB-231*) and one normal-like (*MCF10A*)) exposed to magnetic nanoparticles (*MNP*) for 24 h and 72 h. Controls are free Nucant multivalent pseudopeptide (*N6L*), free doxorubicin (*DOX*) and untreated cells. Each segment represents the analysis of n = 3 experiments with triplicate wells for each parameter: cell count reduction, lysosomal mass and cell permeability as indicated. Colorimetric gradient ranges from: *dark green* = <15% of maximum value measured; *bright green* = 30%; yellow = 50%; bright orange = 60%; dark orange = 75%; red = >75%. Color-coded map values are normalized using the percentage of the negative controls. *N*/*C* negative control, *P*/*C* positive control.

The multiparametric analysis in absence of hyperthermia showed differences in the cytotoxicity between MNP-induced response and the response of free N6L or DOX at two time points (24 and 72 h). It was found that concentrations up to 100 μg Fe/ml did not trigger MNP-induced cytotoxicity (Figure 6, first column compared to subsequent five columns). Interestingly, the normal-like MCF10A cell model did show a consistent pattern across all MNP in the lysosomal and cell permeability response. Conversely, the MCF7 and MDA-MB-231 showed incremental drug-induced toxicity response when subjected to higher load of MNP; this was particularly evident at the 72 h time point. Interestingly, a different drug-activity response was shown by the MNP when coated with N6L or DOX or both N6L and DOX, with the latter combination inducing an equivalent cytotoxic response in the breast cancer cell lines compared to free DOX at the 72 h time point.

**Figure 7:**
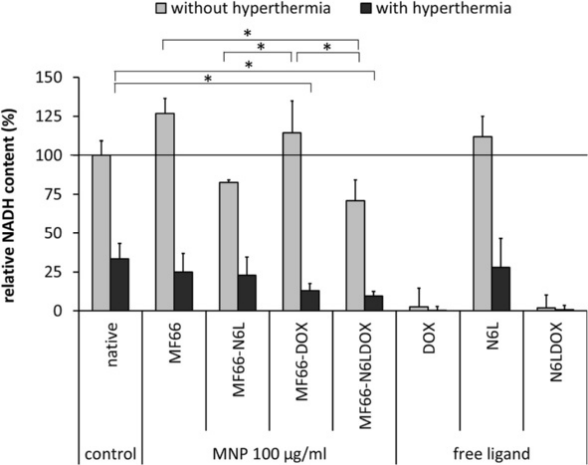
In combination with hyperthermia, functionalized magnetic nanoparticles (*MNP*) were more cytotoxic than non-functionalized *MNP* in *MDA-MB-231* cells. Concentration-dependent reduction of *NADH* content of *MNP* formulations and free ligands at the highest concentration of 100 μg Fe/ml (equivalent to 400 nM Nucant multivalent pseudopeptide (*N6L*) and 4000 nM doxorubicin (*DOX*)) with and without hyperthermia (0 CEM43T90 vs 90 CEM43T90) after 48 h in MDA-MB-231 cells. Means and standard error of the mean of three individual experiments with three parallels each. *Statistically significant at *p* <0.01.

In combination with hyperthermia, MF66-DOX unfolded a higher cytotoxic potential than MF66 and MF66-N6L 48 h after hyperthermia treatment in MDA-MB-231 cells (Figure 7). The combination of N6LDOX on MF66 led to the highest reduction of cell viability 48 h post hyperthermia (*p* <0.01 compared to untreated control cells, *t* test). Importantly, our in vitro experiments revealed that the influence on cell viability of MF66 functionalized with DOX (MF66-DOX and MF66-N6LDOX) was synergistically increased by the hyperthermia treatment, which can trigger the release of DOX. The free DOX was equally cytotoxic with and without hyperthermia to MDA-MB-231 breast cancer cells (Figure 7). Hence, the cytotoxic potential of DOX-functionalized MNP can be triggered by heat to protect healthy cells and tissues.

### Therapeutic temperature dosages were reached in vivo

**Figure 8:**
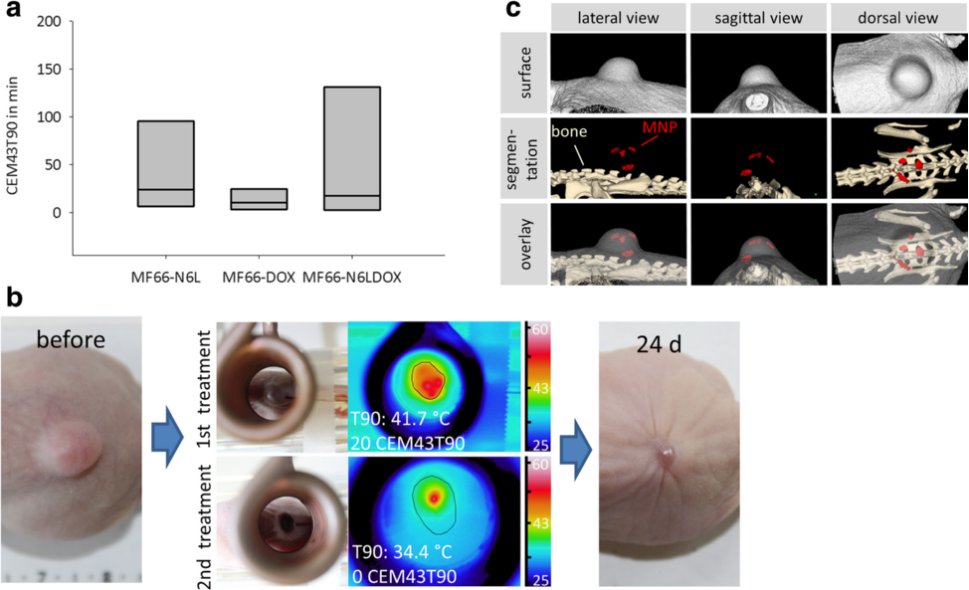
Individual temperature dosages over tumor areas. **a** By using tumor surface temperature during hyperthermia treatment, median temperature dosages were calculated as cumulative equivalent minutes (*CEM43T90*) and displayed as box plots. **b** Example of a treatment sequence within the alternating magnetic field (AMF), the corresponding temperature distribution over the tumor surface and the effect on tumor volume. **c** Intratumoral distribution of magnetic nanoparticles (MNP) (*MF66-N6LDOX*) was determined using micro computed tomography 24 h prior to the first hyperthermia treatment. *N6*L Nucant multivalent pseudopeptide, *DOX* doxorubicin.

The calculated temperature dosages (CEM43T90) of the in vivo hyperthermia treatment showed temperature dosages in the therapeutic regime, but also large variations due to heterogeneity of the temperature distribution. The median CEM43T90 values were 23 minutes for MF66-N6L treated animals, 10 minutes in the MF66-DOX group and 17 minutes for the MF66-N6LDOX animals (Figure 8a). Tumor surface temperatures were monitored with a thermal camera, as shown in Figure 8b. Heterogeneous MNP distribution was confirmed by µCT imaging, here shown exemplary for a mouse injected with MF66-N6LDOX and segmented for bone, the injected MNP and skin (Figure 8c). The exact three-dimensional MNP distribution within the tumor in relation to non-tumor structures (e.g. spinal cord) allowed for a more specific heat generation within the tumor. Safety of the treatment was verified by monitoring blood count parameters, hemoglobin, and white and red blood cells, which did not change over the experimental period (Additional file 4: Figure S3).

### Significant tumor volume reduction following hyperthermia treatment in vivo

**Figure 9:**
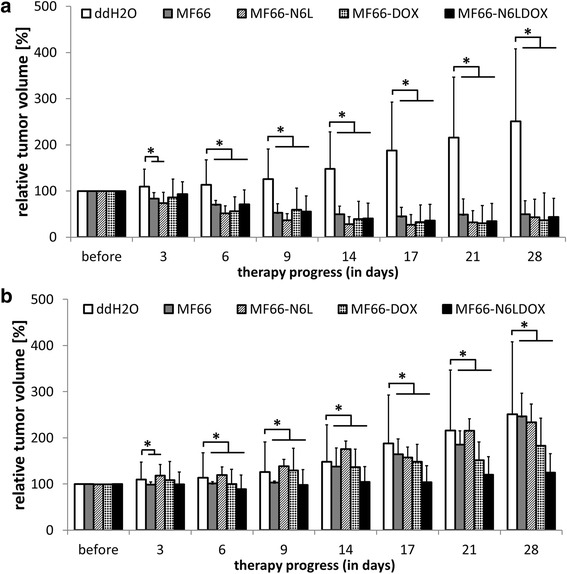
Magnetic hyperthermia treatment with magnetic nanoparticles (MNP) led to a significant reduction in tumor volume compared to untreated animals. Tumor volume was calculated relative to the tumor volume at day 0 (V_t0_) in percent. **a** Tumor volume development after magnetic hyperthermia (60 minutes at *H* = 15.4 kA/m, *f* = 435 kHz) with the different MNP formulations compared to the untreated control (*ddH*_*2*_ *0*, no magnetic hyperthermia). **b** Effect of intratumoral presence of MNP without hyperthermia treatment on tumor volume as well as the *ddH*_*2*_ *0* control (*p ≤0.05, Mann-Whitney U test, treated vs untreated). Means and SD of n ≥6 animals/group.

Tumor growth was strongly inhibited by in vivo magnetic hyperthermia treatment after intratumoral injections of all MNP formulations. For all formulations, we observed an immense reduction of tumor volume to around 40% of the initial tumor volume (V_t0_) over the course of 28 days, while it increased to a mean of 251% of V_t0_ in the untreated control group (ddH_2_O, no AMF treatment, Figure 9a). The relative loss of tumor volume was greatest between day 17 and day 21 of the therapy, while afterwards tumor volume slightly increased by 5–10% during the last week of the experiment.

Hence, the magnetic hyperthermia treatment resulted in comparable tumor volumes at day 28 independent from the MNP formulation: MF66 (50 ± 29% of V_t0_), MF66-N6L (43 ± 39% of V_t0_), MF66-DOX (37 ± 59% of V_t0_), and MF66-N6LDOX (44 ± 41% of V_t0_). A complete tumor regression, i.e. a tumor volume below 20% of V_t0_, varied with 50% (3 out of 6 cases) for MF66, 43% (3 out of 7 cases) for M66-N6L, 71% (5 out of 7 cases) for MF66-DOX, and 33% (2 out of 6 cases) for MF66-N6LDOX-treated mice. In contrast, treatment with MNP alone (without hyperthermia treatment) revealed pronounced differences in the effects of the MNP formulations (Figure 9b). The intratumoral injection of MF66 led to a tumor volume of 246 ± 50% of V_t0_ whereas intratumorally injected MF66-N6L resulted in a tumor volume of 234 ± 138% of V_t0_, comparable to the untreated control group (ddH_2_O, no AMF treatment, 251 ± 164% of V_t0_). Intratumoral MF66-DOX injection slowed down tumor growth considerably and resulted in a final tumor volume of 183 ± 124% of V_t0_. The strongest growth-reducing effect was caused by MF66-N6LDOX, where tumors showed barely any growth with a final tumor volume of 125 ± 61% of V_t0_. Even without hyperthermia the functionalization of MNPs mediated an anti-cancer effect which was highest when the cell internalization moiety and cytotoxic agent were combined. Macroscopically the hyperthermia treatment resulted in the occurrence of eschars over the tumor area and the subsequent loss of tumor volume, whereas these effects were not observed in animals without hyperthermia treatment (Additional file 5: Figure S4).

### Nanoparticles remained within the tumor after MNP application

**Figure 10:**
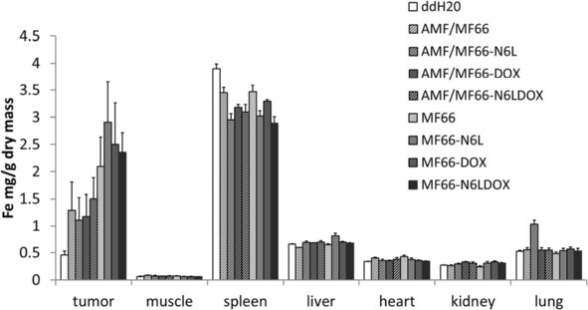
Biodistribution analysis revealed that intratumorally injected magnetic nanoparticles (MNP) were not transported to the organs. Iron content was determined using flame atomic absorption spectrometry after drying and ashing of the tissues and subsequent solubilization of the samples with 65% nitric acid. Means and standard error of the mean of n ≥4 animals/group. *N6L* Nucant multivalent pseudopeptide, *DOX* doxorubicin.

The iron content at the tumor site equaled the expected amount of approximately 2.5 mg Fe per g dry mass for the animals without hyperthermia treatment. A reduction of the iron content was detected for all AMF-treated groups. The hyperthermia treatment led to crust formation which also comprised internalized MNP; thus, during the healing process the crust fell off and decreased detectable iron concentrations. The iron content of the ddH2O-treated animals was attributed to intrinsic iron from tumor vasculature. The iron content in the animal organs 29 days after intratumoral MNP injection was not increased compared to animals that did not receive MNP (Figure 10). This shows that the MNP were not kinetically removed from the tumor site. Only in the group treated with MF66-N6L and hyperthermia did we detect increased iron content in the lung, which did not appear in animals that received MF66-N6L without hyperthermia treatment. Interestingly, in all groups that received MNP, we observed a decrease in the iron content of the spleen of about 25% compared to the untreated control group (Figure 10).

### Increased apoptosis after hyperthermia treatment

**Figure 11:**
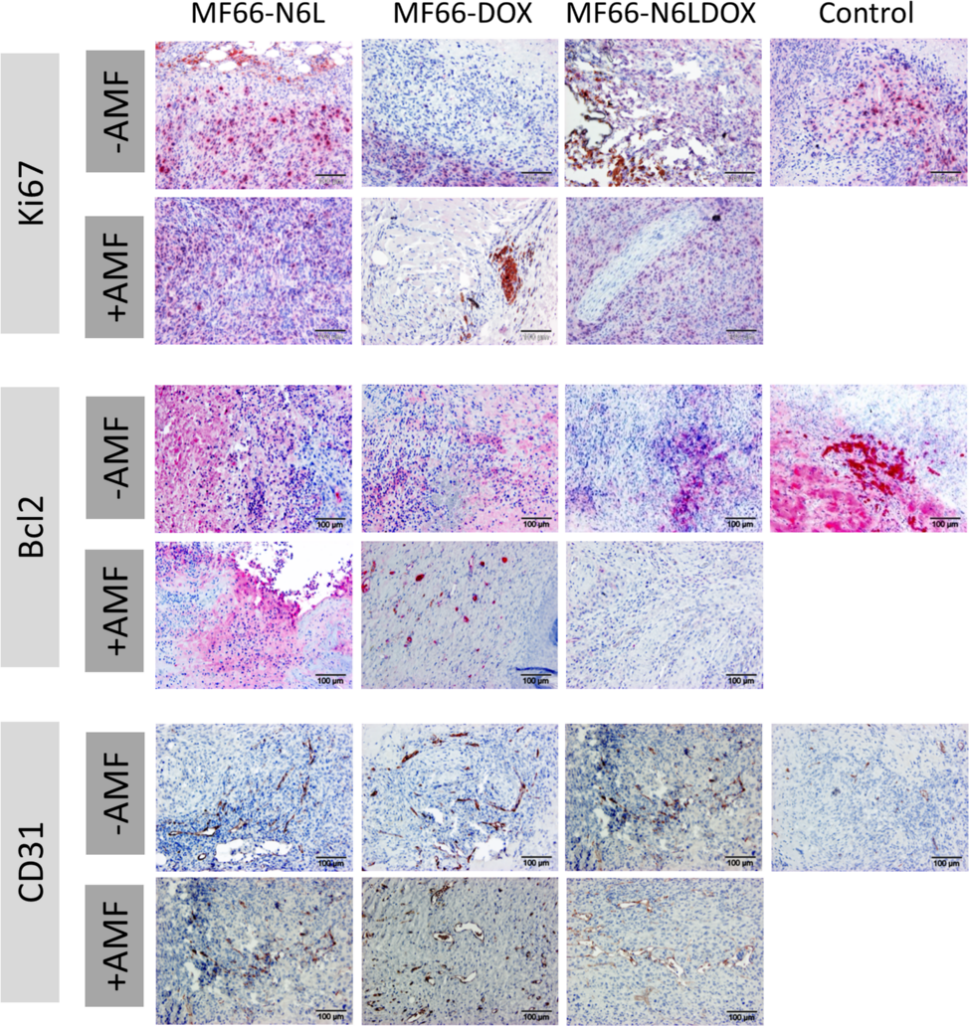
Histological staining for the proliferation marker Ki67, anti-apoptotic protein Bcl2 and tumor vascularization by CD31. Immunohistochemistry of paraffin-embedded formaldehyde-fixed tumor tissue sections stained for expression of Ki67 (*red*), Bcl2 (*red*) and CD31 (*brown*) antigens for animals treated with MF66-Nucant multivalent pseudopeptide (*MF66*-*N6L*), MF66-doxorubicin (*MF66*-*DOX*) or *MF66-N6LDOX* with or without alternating magnetic field (*AMF*) exposure as well as the ddH_2_O controls (*Control*) without AMF treatment. One representative image for each group is shown. Scale bar = 100 μm.

Proliferation (Ki67 expression) of cells in excised tumors was reduced after magnetic hyperthermia, but a statistically significant effect was not detected (Additional file 6: Figure S5, *p* = 0.37). Interestingly, among all formulations, MF66-DOX inhibited the proliferative activity even without magnetic hyperthermia (Figure 11). On the other hand, MF66-N6L and MF66-N6LDOX led to a slight increase of proliferation, and decreased proliferation was observed when magnetic hyperthermia was applied. Apoptosis was also significantly triggered after magnetic hyperthermia, but not after MNP application alone (Additional file 6: Figure S5, *p* = 0.008), as suggested by the reduction of Bcl2 expression. Significantly different Ki67 or Bcl2 expression was not identified in relation to the various MF66 formulations and magnetic hyperthermia. No differences in vascularization (CD31 expression) were detected when the different MNP were injected into the tumors, independent of magnetic hyperthermia treatment.

## Discussion

The SAR (461–900 W/g Fe) and ILP (4.5–8.7 nHm^2^/kg) values of the MNP formulations are among the top values reported in the literature [7, 32, 33] and correspond to previously described features of heating potential and structure [34]. In water suspension the functionalization of the MNP lowered the SAR and ILP by up to 50% compared to non-functionalized MF66. Our results show size-dependence. The smaller the hydrodynamic diameter of the MNP, the higher the SAR was in water suspension. MNP were found to be immobilized to membranes or in intracellular vesicles leading to inhibition of Brownian motion as the heating mechanism; therefore, the heating potential of MNP in an immobilized state is more relevant [35, 36]. We showed that after PVA immobilization, ILP values of functionalized MNP (3.0–3.6 nHm^2^/kg) were almost as high as the values of non-functionalized MF66 (4.7 nHm^2^/kg), supporting the theory that Neel relaxation is the predominant heating mechanism. Importantly, ILP values were still high compared to values for other nanoparticles reported in the literature [14], rendering the MNP with these exceptional physicochemical properties suitable candidates for in vivo magnetic heating applications.

The particles were stable in water as well as in cell culture medium (complete DMEM). Stability of the nanoparticles is supported by the strongly negative ζ-potential of −30 mV or lower, provided by carboxylic acid functions of the DMSA coating. Values below −30 mV or above +30 mV promote strong electrostatic repulsion between particles, whereas values near 0 mV lead to particle flocculation and nanoparticle clusters [21]. The ζ-potential values for functionalized MF66 were slightly higher compared to bare MF66 due to the quenching of negative charges by the adsorbed molecules. In the case of N6L, the quenching was higher because N6L is highly positively charged. However, each formulation displays enough negative charge to be stable at a physiological pH.

N6L and DOX were released from the MNP slowly in the presence of salts over a period of 80 h, whereas the cellular uptake of the MNP was much faster within the first 24 h. Therefore, the intratumoral application was completed 24 h prior to the initial hyperthermia treatment to ensure that most of the nanoparticles will prospectively enter the cells before releasing DOX or N6L. The differences in the release profiles of DOX and N6L can be attributed to the positively charged lysine and arginine residues in N6L and its stronger electrostatic interactions with the DMSA coating [15]. More N6L molecules were released in complete DMEM than in PBS, whereas a higher release was observed in PBS than in complete DMEM for the DOX samples. Hence, the main parameters influencing the release are ionic strength and concentration of other molecules in the environment of the MNP. Cations or other positively charged biomolecules might displace DOX or N6L in the tumor in vivo. Furthermore, the acidic intracellular pH might also play a role in the intracellular release.

Interestingly, substantially higher amounts of MF66-N6L and MF66-N6LDOX were internalized by the cells compared to MF66 and MF66-DOX as seen for the Prussian blue staining. Even though the mechanism of MNP internalization is not fully understood, it is assumed that the multivalent pseudopeptide N6L participates in the internalization of MNP [18]. Therefore, it is postulated that higher levels of N6L-functionalized MNP accumulate in vivo as well. Accumulation and/or internalization of MNP is crucial towards a successful therapy combining hyperthermia and local chemotherapy in vivo [37]. These features make the MNP particularly useful for intratumoral application, where the biomolecules are allowed to desorb from the MNP before starting the magnetically induced heating process.

The amount of DOX conjugated to the MNP was highly cytotoxic to cells when applied as free DOX. When conjugated to the MNP, the same molar amount of DOX had almost no influence on cell viability at a short exposure time of 24 h. Following exposure for 72 h the cytotoxic potential of MF66-N6LDOX was comparable to free DOX in the breast cancer cell lines. Conversely, minimal decrease in cell count was recorded in the MCF10A cell line. Hence, it is of interest to point out that in the conjugated state the systemic cytotoxicity of DOX was time-dependent. Release occurred either by longer incubation times, as shown by live cell imaging, or by heat treatment, leading to a synergistic effect of heat and DOX. Consequently, MF66-DOX and MF66-N6LDOX mediated more damage to cells than MF66 or hyperthermia only.

The MNP presented in this study are highly capable tools for in vivo hyperthermia treatment. Independent of the MNP formulation, magnetic hyperthermia led to hyperthermic temperatures in the tumor area which resulted in significantly reduced tumor volumes compared to the untreated control, and in 50% there was macroscopically complete tumor regression. In fact, the remaining tumor volume after 28 days was due more to cicatricle tissue at the tumor site and less to the actual tumor mass. A distinct effect of the functionalization on tumor growth was observed in vivo. Here, MF66-DOX diminished tumor growth also in the absence of hyperthermia treatment, whereas MF66-N6L did not to the same extent. The strongest growth-reducing effect was mediated by MF66-N6LDOX, where the tumors remained at their initial tumor volume over the period of 28 days, indicating a synergistic effect of the multifunctionalization in vivo, supporting our in vitro results. The MF66-N6L particles proved capable cell internalization agents. Higher concentration of N6L molecules on the MNP surface would further increase their antitumoral effect (unpublished data).

With consideration of the tumor volumes after AMF treatment, we assume that the effects mediated by functionalization were masked by the stronger effects of the heat treatment alone, which was highly effective in subcutaneous xenografts. The presence of high local concentrations of chemotherapeutic agents in the tumoral region is important to avoid tumor relapse after hyperthermia treatment, especially in tumor areas where temperature under-dosage is more likely to occur [38].

Intratumoral MNP application showed no systemic side effects and demonstrated good biocompatibility for the DOX-functionalized and N6L-functionalized MNP by an unaltered blood composition. The biodistribution analysis confirmed only negligible release of intratumorally injected MNP from the tumor area into other body compartments. Therefore, several AMF treatments can be conducted without re-injection of MNP, and magnetic hyperthermia will not affect any organs if MNP are administered intratumorally [10], in contrast to intravenous injection where most of the injected MNP are deposited in the liver and the spleen [39]. Interestingly, we observed a decrease in iron content in the spleen upon intratumoral injection of MNP. To our knowledge such an effect has not been reported before. Yallapu et al. [40] observed little uptake in the liver but no change in the iron content of the spleen after intratumoral injection of curcumin-loaded iron oxide nanoparticles.

Immunohistochemistry showed that MF66-DOX themselves affected the tumor cells and decreased their viability by increasing apoptosis and inhibiting cell proliferation. MF66-N6L and MF66-N6LDOX were almost inert without hyperthermia treatment, but all MNP mediated strong cytotoxic effects in conjunction with hyperthermia, revealing an additive effect of ligand and heat.

We believe that the proposed MNP formulations have high potential for translation to clinical practice. They are basically made up of components previously approved by the Food and Drug Administration (FDA) (particularly iron oxide and DOX). The intratumoral application of the MNP using stereotactic methods commonly used in radiology would allow a very local exposure of the human body, a strategy that distinctly minimizes the known side effects of DOX.

## Conclusions

The MNP presented in this study are well-suited for hyperthermia treatments. We demonstrated in vitro that N6L functionalization improved intracellular uptake and DOX functionalization mediated additional cytotoxicity compared to non-functionalized MNP. In vivo the MNP led to an immense reduction in tumor volume and almost complete regression in many cases. We observed that proliferative activity of tumor cells was abolished most prominently by MF66-DOX MNP. The functionalized MNP presented here offer new prospects for optimizing tumor treatment by magnetic hyperthermia using MNP with high heating potential. The additional electrostatic conjugation with N6L and DOX will prospectively increase the MNP load in cells and further improve their cell inactivation potential. The MNP presented here are particularly suitable for intratumoral application of magnetic materials. With this technique, particularly early stages of breast cancer with solitary tumors and negative lymph node status could be treated, which are increasingly detected as a result of implementation of improved diagnostic methods in radiology.

## Additional files

Additional file 1:
**Description of supplementary method.**
Additional file 2: Figure S1. Magnetic nanoparticles (MNP) are stable in water and do not agglomerate in complete cell culture medium. **a** All particle formulations are stable in water for at least 10 days at a concentration of 0.1 g/L (no increase of the hydrodynamic sizes observed over time). **b** In order to simulate biological conditions, the MNP were transferred from water to complete DMEM (gray vertical line) at a final concentration of 0.1 g/L. The hydrodynamic size increased after transfer to complete DMEM as proteins adsorb to the MNP. Here, a complete agglomeration of the MNP was not observed; therefore, the MNP are stable in complete DMEM for at least 48 h. Additional file 3: Figure S2. Subcellular localization of MF66 magnetic nanoparticles (MNP). Visualization of MDA-MB-231 cells after 24 h incubation with MF66 MNPs or untreated cells in bright field, fluorescence microscopy, and merged images, respectively. Lysotracker® Red displays localization of lysosomes in cells. A substantial fraction of the red fluorescence from the LysoTracker® dye co-localizes with the brown spots, which represent internalized MNP. Scale bar = 10 nm. Additional file 4: Figure S3. Application of magnetic nanoparticles (MNP) with or without hyperthermia treatment did not alter the blood composition, indicating good biocompatibility of the therapeutic modality. The number of white blood cells (*10^3^/μl), red blood cells (*10^6^/μl), and the amount of hemoglobin (g/dl) are displayed for animals treated with MF66-N6L (**a**), MF66-DOX (**b**) or MF66-N6LDOX (**c**) with or without alternating magnetic field (AMF) treatment in comparison to untreated control animals before, and at 2 and 4 weeks after MNP application. *Black lines* refer to reference values (Harlan Laboratories, Venray, The Netherlands; http://www.harlan.com). Additional file 5: Figure S4. Magnetic hyperthermia treatment of superficial tumors leads to crust building and disappearance or volume reduction of tumors. Photographs of animals at the indicated time points qualitatively show the development of tumors between day 0 and day 28 of the therapy. Animals were either treated or not treated with magnetic hyperthermia after intratumoral injection of MF66-N6L, MF66-DOX or MF66-N6LDOX. Shown are three representative animals per time point and group (n = 6/group). *Arrows* point to subcutaneous tumors. Additional file 6: Figure S5. Error bars of the mean expression levels indicate a significant difference in apoptosis (Bcl2) after hyperthermia treatment. Histological slides of the tumor tissues were grouped into five categories based on the expression levels of tumors for (**a**) Ki67 and (**b**) Bcl2. The Mann-Whitney U test was conducted for magnetic nanoparticles (MNP) with and without hyperthermia based on the mean expression level. The significance level was set at *p* ≤0.05.

## Abbreviations

AMF: Alternating magnetic field
DMEM: Dulbecco’s modified Eagle’s medium
DMSA: Dimercaptosuccinic acid
DMSO: Dimethyl sulfoxide
DOX: Doxorubicin
FBS: Fetal bovine serum
ILP: Intrinsic loss power
MNP: Magnetic nanoparticles
N6L: Nucant multivalent pseudopeptide
N6L-AF546: Nucant multivalent pseudopeptide labeled with Alexa Fluor 546
PBS: Phosphate-buffered saline
SAR: Specific absorption rate
SEM: Standard error of the mean
