# Supplementary material for: Efficient treatment of breast cancer xenografts with multifunctionalized iron oxide nanoparticles combining magnetic hyperthermia and anti-cancer drug delivery
Source: Breast Cancer Res. 2015 May 13;17(1):66. doi: 10.1186/s13058-015-0576-1 (PMC4451751; doi:10.1186/s13058-015-0576-1)
Supplement: Additional file 1: — Description of supplementary method. [file 13058_2015_576_MOESM1_ESM.docx]

**Efficient treatment of breast cancer xenografts with multifunctionalized iron oxide nanoparticles combining magnetic hyperthermia and anti-cancer drug delivery**

Susanne Kossatz^1^ Email: [susanne.kossatz@outlook.de](mailto:susanne.kossatz@outlook.de)

Julia Grandke^1^ Email: [julia.grandke@med.uni-jena.de](mailto:julia.grandke@med.uni-jena.de)

Pierre Couleaud^2,5^ Email: [pierre.couleaud@imdea.org](mailto:pierre.couleaud@imdea.org)

Alfonso Latorre^2,5^ Email: [alfonso.latorre@imdea.org](mailto:alfonso.latorre@imdea.org)

Antonio Aires^2,5^ Email: [antonio.aires@imdea.org](mailto:antonio.aires@imdea.org)

Kieran Crosbie-Staunton^3^ Email: [crosbiek@tcd.ie](mailto:crosbiek@tcd.ie)

Robert Ludwig^1^ Email: [robert.ludwig@med.uni-jena.de](mailto:robert.ludwig@med.uni-jena.de)

Heidi Dähring^1^ Email: [heidi.daehring@med.uni-jena.de](mailto:heidi.daehring@med.uni-jena.de)

Volker Ettelt^1^ Email: [v_ettelt@web.de](mailto:v_ettelt@web.de)

Ana Lazaro-Carrillo^4^ Email: [ana.lazaro@uam.es](mailto:ana.lazaro@uam.es)

Macarena Calero^2,4^ Email: [[macarena.calero@uam.es](mailto:macarena.calero@uam.es)](mailto:susanne.kossatz@outlook.de)

Maha Sader^6^ Email: [sader.maha@gmail.com](mailto:sader.maha@gmail.com)

José Courty^6^ Email: [courty@u-pec.fr](mailto:courty@u-pec.fr)

Yuri Volkov^3,7^ Email: [YVOLKOV@tcd.ie](mailto:YVOLKOV@tcd.ie)

Adriele Prina-Mello^3,7^ Email: [PRINAMEA@tcd.ie](mailto:PRINAMEA@tcd.ie)

Angeles Villanueva^2,4^ Email: [angeles.villanueva@uam.es](mailto:angeles.villanueva@uam.es)

Álvaro Somoza^2,5^ Email: [alvaro.somoza@imdea.org](mailto:alvaro.somoza@imdea.org)

Aitziber L. Cortajarena^2,5^ Email: [aitziber.lopezcortajarena@imdea.org](mailto:aitziber.lopezcortajarena@imdea.org)

Rodolfo Miranda^2,5^ Email: [rodolfo.miranda@imdea.org](mailto:rodolfo.miranda@imdea.org)

Ingrid Hilger^1^ Email: [ingrid.hilger@med.uni-jena.de](mailto:ingrid.hilger@med.uni-jena.de)

^1^Institute for Diagnostic and Interventional Radiology, Jena University Hospital – Friedrich Schiller University Jena, D-07740 Jena, Germany

^2^Instituto Madrileño de Estudios Avanzados en Nanociencia (IMDEA Nanociencia), Campus Universitario de Cantoblanco, 28049 Madrid, Spain

^3^School of Medicine, Trinity College Dublin, Ireland

^4^Departamento de Biología, Universidad Autónoma de Madrid, Cantoblanco, 28049 Madrid, Spain

^5^Unidad Asociada de Nanobiotecnología CNB-CSIC & IMDEA Nanociencia, Campus Universitario de Cantoblanco, 28049 Madrid, Spain

^6^Laboratoire CRRET, Université Paris EST Créteil, 61 Avenue du Général de Gaulle, 94010 Créteil, France

^7^CRANN, Trinity College Dublin, Ireland

Corresponding authors: Dr. Julia Grandke and Prof. Dr. Ingrid Hilger, Institute for Diagnostic and Interventional Radiology , Jena University Hospital – Friedrich Schiller University Jena, D-07740 Jena, Phone: 0049-(0)3641-9325921, Fax: 0049-(0)3641-9325922, Email: [julia.grandke@med.uni-jena.de](mailto:julia.grandke@med.uni-jena.de) and ingrid.hilger@med.uni-jena.de

***Supplementary data***

***Supplementary method description***

**Subcellular localization of MNPs**

To determine the intracellular localization of MNP, MDA-MB-231 cells growing on a glass coverslip were incubated with MNP (100 µg Fe/ml) for 24 h. Then, cells were labeled with the lysosomotropic fluorophore LysoTracker^®^ Red DND-99 (Molecular Probes, Eugene, Oregon, USA) at 50 nM in culture medium at 37 °C for 30 min. After washing, cells were observed immediately under bright light illumination or fluorescence (green excitation filter) to detect the internalized MNP and the emission of LysoTracker^®^, respectively.

**Determination of temperature dosage**

The calculation of temperature dosages, which was applied in *in vitro* hyperthermia experiments, was performed by measuring the temperature profile of a dummy 96-well-plate. The dummy was filled with 100 µl cell culture media/well (DMEM / RPMI 1640; Gibco®, Paisley, UK*)* and cultured at 37 °C for 2 h. Subsequently, the dummy was heated at 43 °C or 46 °C for 30 min and the temperature within a well was measured continuously with a thermal probe (Optocon AG, Dresden, Germany). Determination of the temperature at 43 °C and 46 °C with cells seeded into the wells showed the same temperature profile, indicating that the thermal capacity of cells does not to alter the temperature profile.

The temperature profile was fitted with a square/cubic polynomial and integrated from 0 to 30 min (0 to 1800 s) to determine the applied thermal dosage in [°C*s]. The calculation of the thermal dosage of 30 min 43 °C resulted in 6359 [°C*s]; 30 min at 46°C in 12600 [°C*s].

The calculation of the thermal dosage does not consider the exponential impact of heating onto cell viability. Therefore, it is common to calculate the thermal dosage applied into cumulative equivalent minutes at 43 °C (CEM43T90). The equation used was postulated by [[31](#_ENREF_31)]. :

$${CEM}_{43}=t*R^{43^{\circ}C-T}$$

with: $R=\left\{ \begin{aligned} 0.25 T \leq43^{\circ}C \\ 0.5 T \geq43^{\circ}C \end{aligned} \right\}$
 *t* = time

 *T* = temperature

For calculation of the CEM43T90 over 30 minutes the CEM43T90 for *T* at *t* had to be summed up for all values of *T*:

$${}_{T_{0}}^{T}{{CEM}_{43}}=\sum_{T_{0}}^{T} t*R^{43^{\circ}C-T}$$

Using this equation resulted in 6 CEM43T90 for 30 min of hyperthermia treatment at 43 °C and 90 CEM43T90 for 30 min treatment at 46 °C. Therefore 30 min at 46 °C was chosen as the therapeutic temperature regime for the *in vitro* experiments.

**Immunohistology**

Before staining, the 3-μm paraffin-embedded sections from explanted tumors were deparaffinised and antigens were retrieved by microwave treatment (25 min, 325 W), followed by avidin/biotin blocking (in case of Ki67 and Bcl2 staining) or peroxidase blocking (for CD31 staining). Between incubation steps, slides were rinsed with 0.1 % TBS-T buffer. At the end, all slides were counterstained with haematoxylin (Sigma-Aldrich, Steinheim, Germany) and covered with Faramount (DAKO, Glostrup, Denmark).

*Ki67*

Immunohistological analysis of Ki67 was performed by utilization of a primary monoclonal anti-Ki67 antibody (Abcam, Cambridge, UK, 1:500 dilution). A polyclonal goat anti-rabbit IgG (H+L)-Biotin-antibody (Dianova, Hamburg, Germany, 1:2250 dilution) was used as secondary antibody. For detection, a streptavidin-AP conjugate (Southern Biotech, Birmingham, USA, 1:75 dilution) and the REAL™ Detection System Alkaline Phosphatase/RED (K5005, DAKO, Glostrup, Denmark) were applied.

*Bcl2*

Tumor sections were also stained for Bcl2 to determine the effects of hyperthermia treatment on apoptosis induction. Here, a primary antibody, monoclonal mouse anti-human Bcl2 (1:500 dilution, Dako, Hamburg, Germany), was incubated for 1 h at room temperature. For secondary antibody incubation and detection the REAL™ Detection System Alkaline Phosphatase/RED (K5005, DAKO, Glostrup, Denmark) was applied.

*CD31*

To evaluate tumor vascularization, vessels were stained for CD31 antigen expression. The primary antibody, a polyclonal rabbit anti-CD31 (1:500 dilution, Abcam, Cambridge, UK), was incubated for 30 min at room temperature. Secondary antibody incubation and detection were carried out as instructed by the manufacturer using the Dako EnVision™+ System-HRP (DAB) (K4010, Dako,Hamburg, Germany).
