# Supplementary figures and images for: Efficient treatment of breast cancer xenografts with multifunctionalized iron oxide nanoparticles combining magnetic hyperthermia and anti-cancer drug delivery
Source: Breast Cancer Res. 2015 May 13;17(1):66. doi: 10.1186/s13058-015-0576-1 (PMC4451751; doi:10.1186/s13058-015-0576-1)

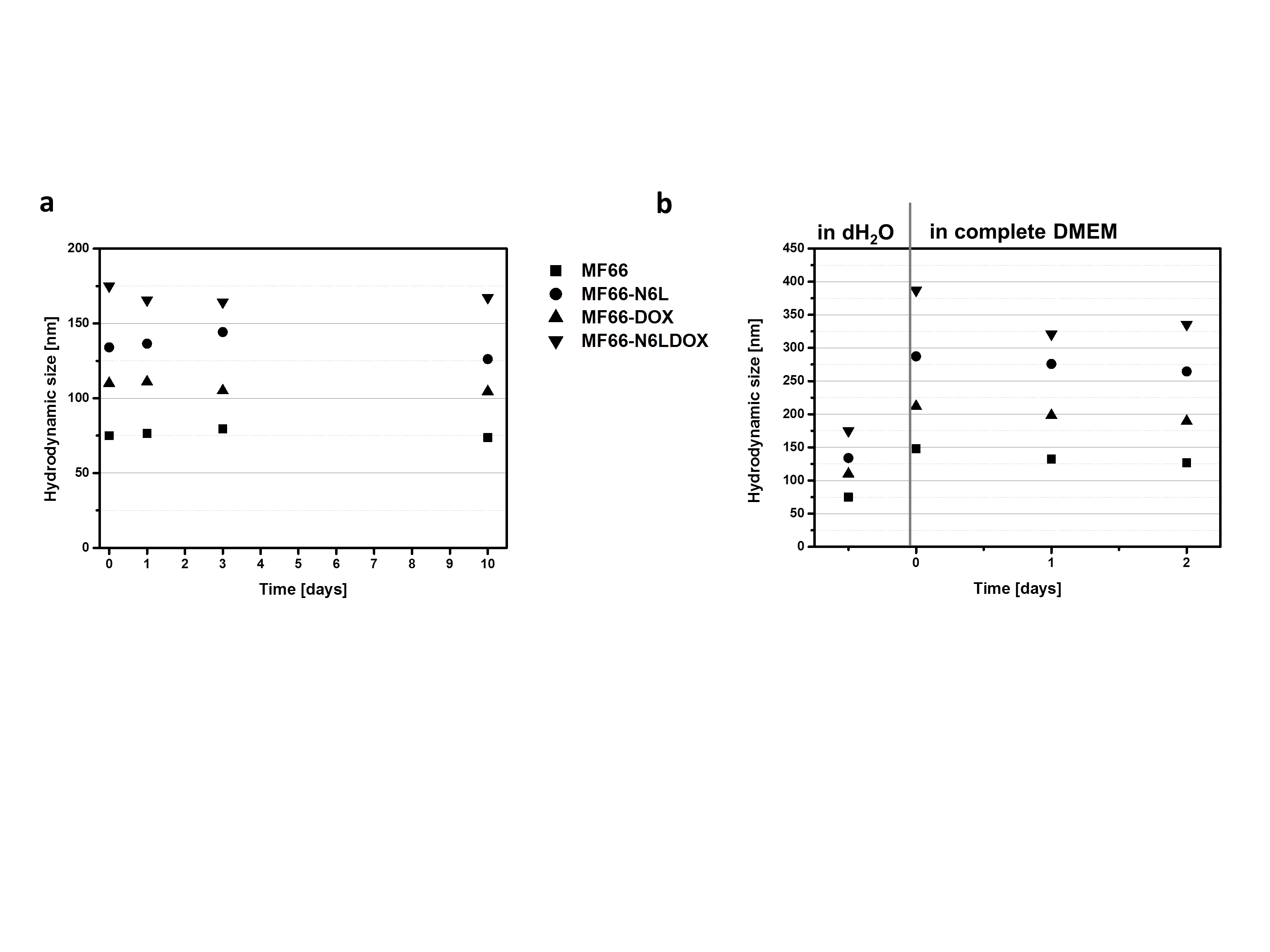

Supplement: Additional file 2: Figure S1. — Magnetic nanoparticles (MNP) are stable in water and do not agglomerate in complete cell culture medium. a All particle formulations are stable in water for at least 10 days at a concentration of 0.1 g/L (no increase of the hydrodynamic sizes observed over time). b In order to simulate biological conditions, the MNP were transferred from water to complete DMEM (gray vertical line) at a final concentration of 0.1 g/L. The hydrodynamic size increased after transfer to complete DMEM as proteins adsorb to the MNP. Here, a complete agglomeration of the MNP was not observed; therefore, the MNP are stable in complete DMEM for at least 48 h. [file 13058_2015_576_MOESM2_ESM.tiff]

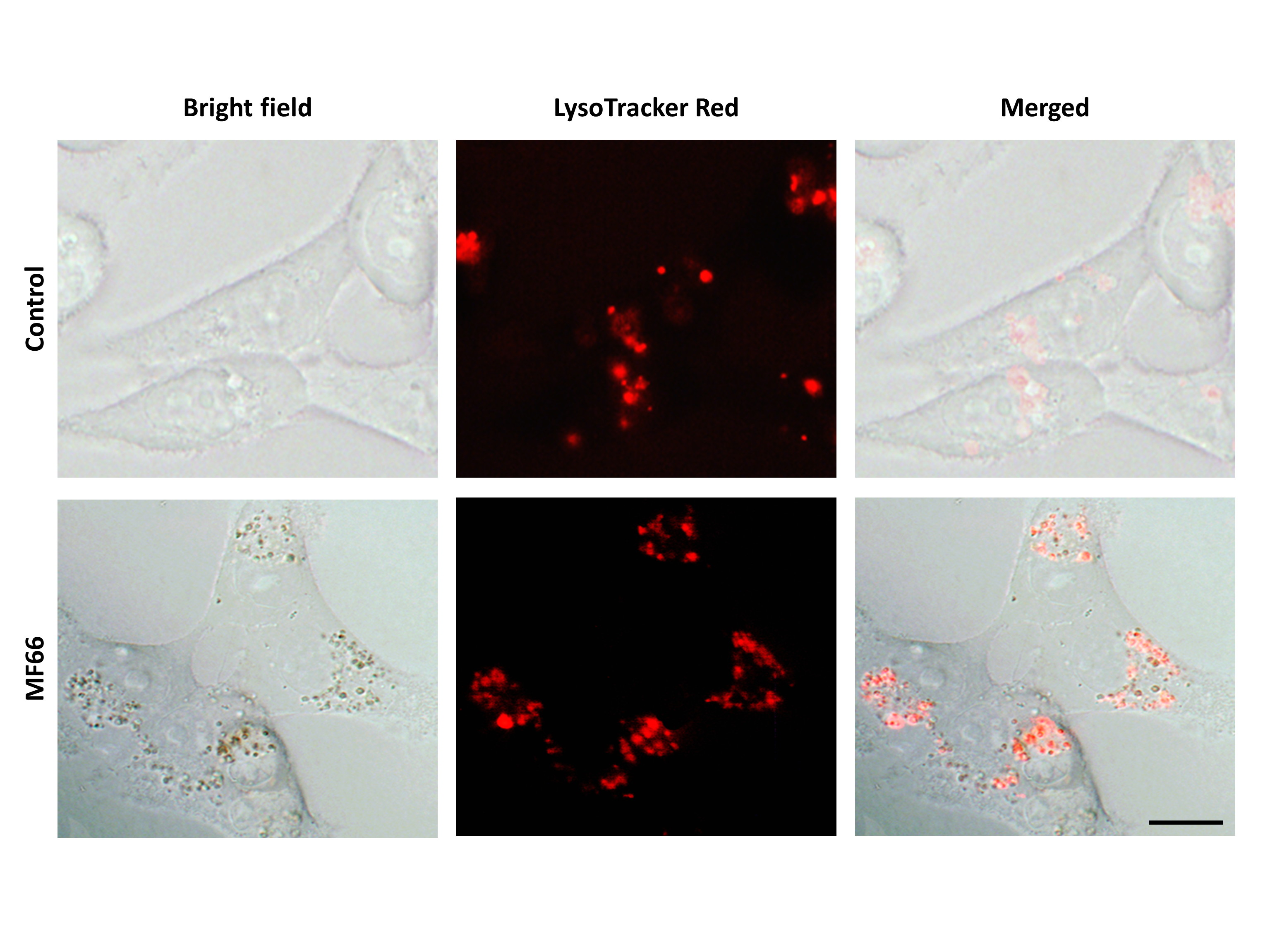

Supplement: Additional file 3: Figure S2. — Subcellular localization of MF66 magnetic nanoparticles (MNP). Visualization of MDA-MB-231 cells after 24 h incubation with MF66 MNPs or untreated cells in bright field, fluorescence microscopy, and merged images, respectively. Lysotracker® Red displays localization of lysosomes in cells. A substantial fraction of the red fluorescence from the LysoTracker® dye co-localizes with the brown spots, which represent internalized MNP. Scale bar = 10 nm. [file 13058_2015_576_MOESM3_ESM.tiff]

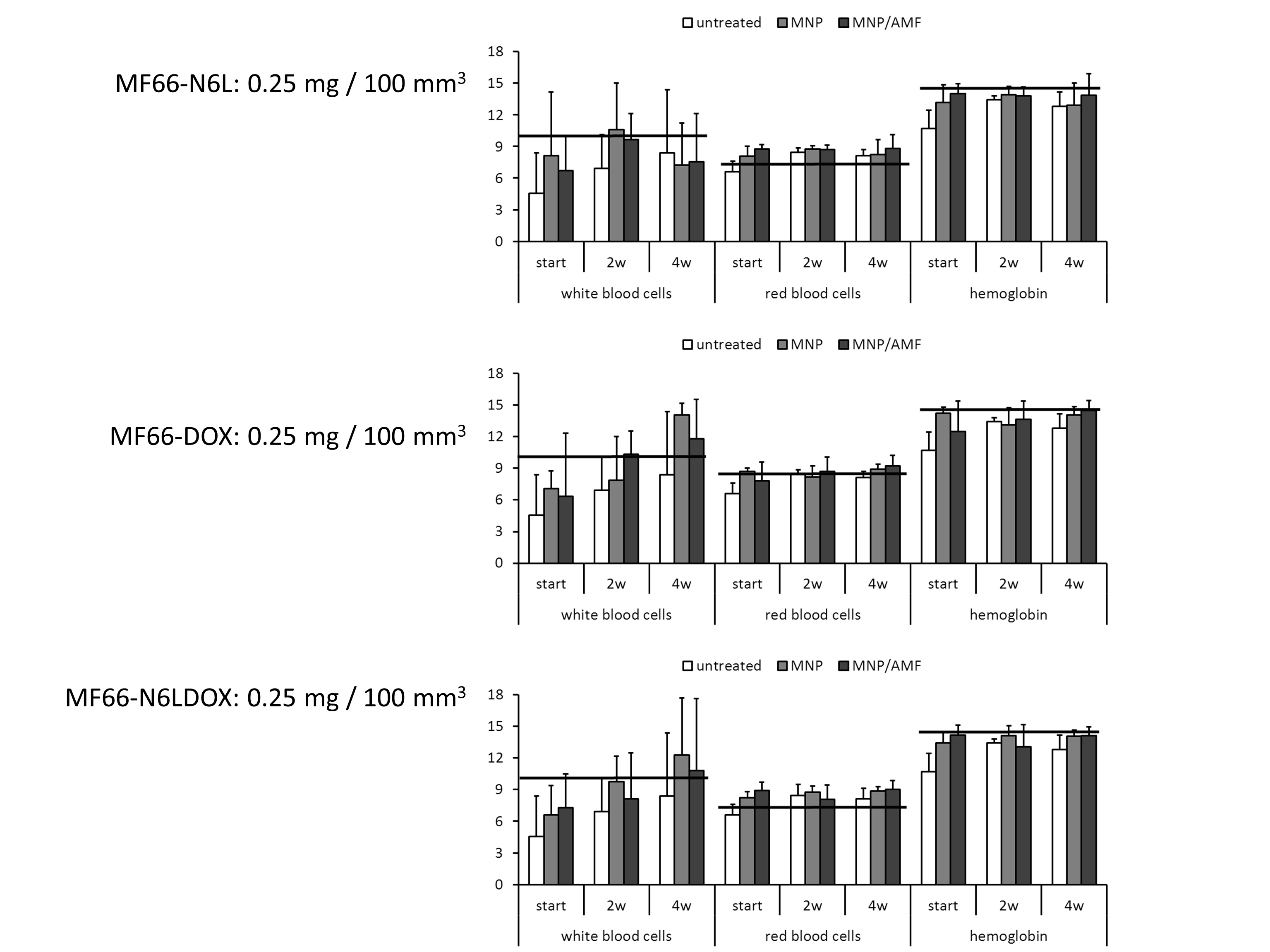

Supplement: Additional file 4: Figure S3. — Application of magnetic nanoparticles (MNP) with or without hyperthermia treatment did not alter the blood composition, indicating good biocompatibility of the therapeutic modality. The number of white blood cells (*103/μl), red blood cells (*106/μl), and the amount of hemoglobin (g/dl) are displayed for animals treated with MF66-N6L (a), MF66-DOX (b) or MF66-N6LDOX (c) with or without alternating magnetic field (AMF) treatment in comparison to untreated control animals before, and at 2 and 4 weeks after MNP application. Black lines refer to reference values (Harlan Laboratories, Venray, The Netherlands; http://www.harlan.com). [file 13058_2015_576_MOESM4_ESM.tiff]

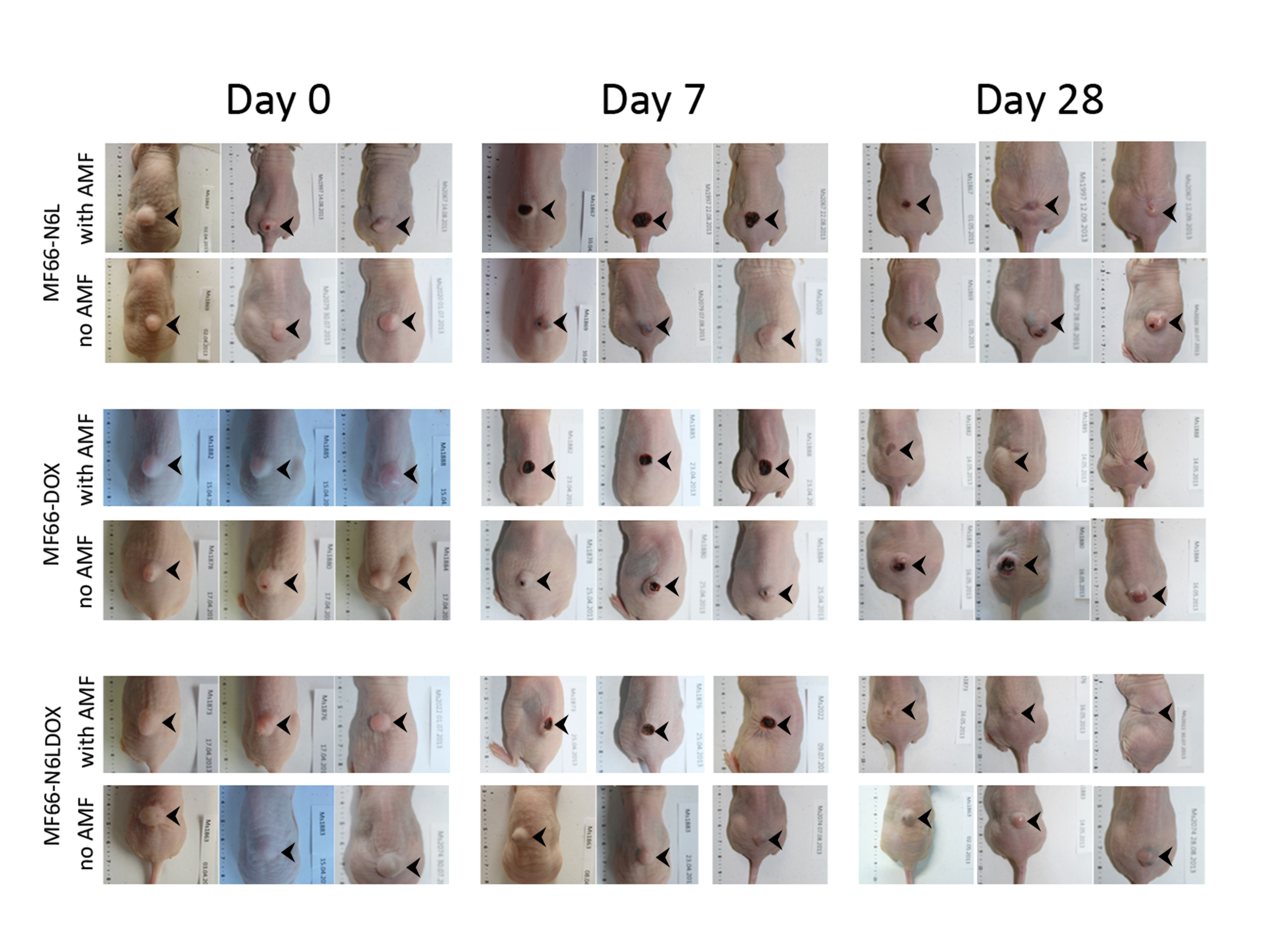

Supplement: Additional file 5: Figure S4. — Magnetic hyperthermia treatment of superficial tumors leads to crust building and disappearance or volume reduction of tumors. Photographs of animals at the indicated time points qualitatively show the development of tumors between day 0 and day 28 of the therapy. Animals were either treated or not treated with magnetic hyperthermia after intratumoral injection of MF66-N6L, MF66-DOX or MF66-N6LDOX. Shown are three representative animals per time point and group (n = 6/group). Arrows point to subcutaneous tumors. [file 13058_2015_576_MOESM5_ESM.tiff]

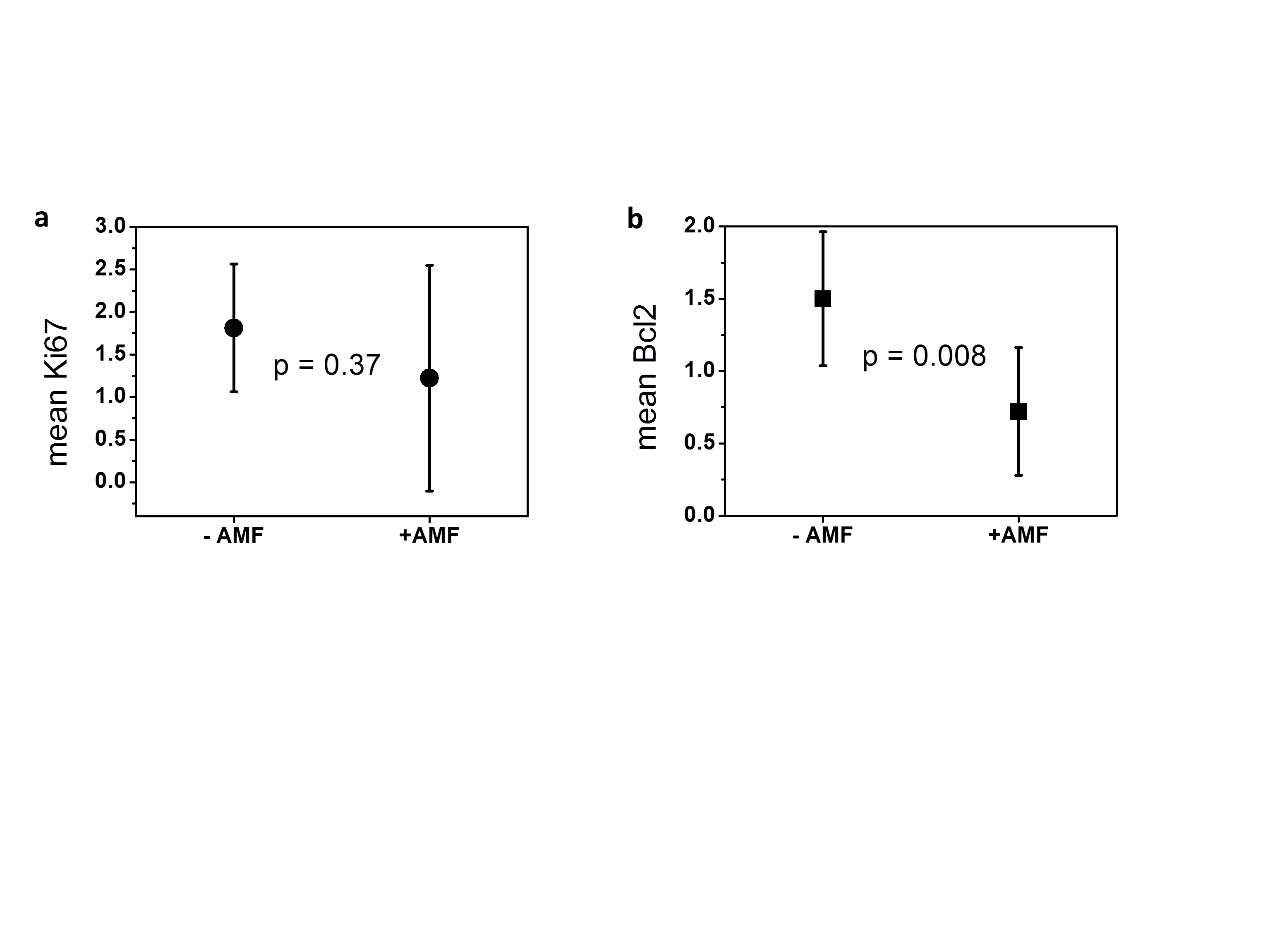

Supplement: Additional file 6: Figure S5. — Error bars of the mean expression levels indicate a significant difference in apoptosis (Bcl2) after hyperthermia treatment. Histological slides of the tumor tissues were grouped into five categories based on the expression levels of tumors for (a) Ki67 and (b) Bcl2. The Mann-Whitney U test was conducted for magnetic nanoparticles (MNP) with and without hyperthermia based on the mean expression level. The significance level was set at p ≤0.05. [file 13058_2015_576_MOESM6_ESM.tiff]
